# Comparative analysis of virus-derived small RNAs within cassava (*Manihot esculenta* Crantz) infected with cassava brown streak viruses

**DOI:** 10.1016/j.virusres.2016.01.015

**Published:** 2016-04-02

**Authors:** Emmanuel Ogwok, Muhammad Ilyas, Titus Alicai, Marie E.C. Rey, Nigel J. Taylor

**Affiliations:** a National Crops Resources Research Institute, Namulonge, P.O Box 7084, Kampala, Uganda; b Institute for International Crop Improvement, Donald Danforth Plant Science Center, St. Louis, MO 63132, USA; c School of Molecular and Cell Biology, University of the Witwatersrand, Private Bag 3, P.O Wits 2050, Johannesburg, South Africa

**Keywords:** Deep sequencing, cassava brown streak disease, cassava, virus-derived small RNA

## Abstract

Infection of plant cells by viral pathogens triggers RNA silencing, an innate antiviral defense mechanism. In response to infection, small RNAs (sRNAs) are produced that associate with Argonaute (AGO)-containing silencing complexes which act to inactivate viral genomes by posttranscriptional gene silencing (PTGS). Deep sequencing was used to compare virus-derived small RNAs (vsRNAs) in cassava genotypes NASE 3, TME 204 and 60444 infected with the positive sense single-stranded RNA (+ssRNA) viruses cassava brown streak virus (CBSV) and Ugandan cassava brown streak virus (UCBSV), the causal agents of cassava brown streak disease (CBSD). An abundance of 21–24 nt vsRNAs was detected and mapped, covering the entire CBSV and UCBSV genomes. The 21 nt vsRNAs were most predominant, followed by the 22 nt class with a slight bias toward sense compared to antisense polarity, and a bias for adenine and uracil bases present at the 5′-terminus. Distribution and frequency of vsRNAs differed between cassava genotypes and viral genomes. In susceptible genotypes TME 204 and 60444, CBSV-derived sRNAs were seen in greater abundance than UCBSV-derived sRNAs. NASE 3, known to be resistant to UCBSV, accumulated negligible UCBSV-derived sRNAs but high populations of CBSV-derived sRNAs. Transcript levels of cassava homologues of AGO2, DCL2 and DCL4, which are central to the gene-silencing complex, were found to be differentially regulated in CBSV- and UCBSV-infected plants across genotypes, suggesting these proteins play a role in antiviral defense. Irrespective of genotype or viral pathogen, maximum populations of vsRNAs mapped to the cytoplasmic inclusion, P1 and P3 protein-encoding regions. Our results indicate disparity between CBSV and UCBSV host-virus interaction mechanisms, and provide insight into the role of virus-induced gene silencing as a mechanism of resistance to CBSD.

## Highlights

• The 21-nt virus-derived small RNAs were predominant, followed by the 22-nt class.
• Susceptible cassava genotypes accumulated higher CBSV- than UCBSV-derived small RNA.
• Tolerant cassava genotype accumulated high CBSV- and low UCBSV-derived small RNAs.
• AGO2, DCL2 and DCL4 were differentially regulated in CBSV/UCBSV-infected plants.
• CBSV and UCBSV interact differently in the same host genetic background

## 1 Introduction

Cassava brown streak disease (CBSD) has become an economically important disease of cassava in East and Central Africa, causing significant losses in both yield and quality of cassava storage roots (Legg et al., 2011, Maruthi et al., 2014, Patil et al., 2015). CBSD is caused by the two distinct, but closely related, positive sense single-stranded RNA (+ssRNA) virus species, cassava brown streak virus (CBSV) and Ugandan cassava brown streak virus (UCBSV). Both CBSV and UCBSV belong to the family *Potyviridae*, genus *Ipomovirus* (Mbanzibwa et al., 2009b, Monger et al., 2001). The viral pathogens are transmitted by whiteflies, *Bemisia tabaci*, Gennadius (Maruthi et al., 2005), and mechanically through grafting (Wagaba et al., 2013, Yadav et al., 2011) and sap inoculation of herbaceous plant species (Lister, 1959, Ogwok et al., 2010). The viruses systemically infect cassava in the field (Kaweesi et al., 2014, Ogwok et al., 2015) causing similar CBSD symptoms on affected cassava plants. CBSV is more aggressive, inducing more rapid and more severe CBSD symptoms than those seen on plants infected with UCBSV alone. Recent studies have shown some cassava varieties, for example NASE 3 (Ogwok et al., 2015) and Kaleso (Maruthi et al., 2014), to be highly resistant to infection by UCBSV (Kabanyolo isolate) but less so to CBSV. The difference in response of cassava varieties to infection with CBSV and UCBSV implies a difference in the mechanism of host-virus interaction between the two virus species.

Plant-infecting viruses are known to trigger RNA interference (RNAi) (Ding, 2010, Llave, 2010), an innate antiviral defense mechanism. This involves critical steps including production of double-stranded RNA (dsRNA), processing of the dsRNA into small interfering RNA (siRNA), followed by sequence-specific targeting and silencing of viral messenger RNA (mRNA) by siRNA-incorporated effector complexes (Seo et al., 2013). The siRNA molecules associate with distinct Argonaute-containing silencing complexes to target and mediate diverse silencing effects on both host and viral genomes (Ding and Voinnet, 2007, Llave, 2010, Xia et al., 2014). During virus–host interactions, virus-specific dsRNAs are generated in a variety of ways, such as activity of virus-encoded RNA polymerase during genomic replication and transcription, or imperfect folding of self-complementary single stranded viral genomic RNA (Ding and Voinnet, 2007). Host-encoded RNA-dependent RNA polymerase (RDR) plays a crucial role in the maintenance of virus-induced gene silencing by converting ssRNAs to dsRNAs for secondary siRNA synthesis (Molnár et al., 2005, Seo et al., 2013). The dsRNAs serve as substrates for Dicer-like (DCL) ribonucleases which cleave the dsRNAs to produce 21–24 nucleotide (nt) virus-derived small RNA (vsRNA) duplexes (Brodersen and Voinnet, 2006, Moissiard and Voinnet, 2006, Zamore et al., 2000). The vsRNAs are then loaded onto Argonaute (AGO) proteins, a component of RNA-induced silencing complexes (RISC), to facilitate cleavage of complementary viral mRNA, consequently silencing the viral genome as a self-defense response of the plant (Ding and Voinnet, 2007, Smith et al., 2011).

Studies in *Arabidopsis thaliana* and other plant species have revealed that the core components involved in plant small RNA biogenesis and the silencing pathways are encoded by multi-protein families (Seo et al., 2013). These are diverse and exhibit functional redundancy (Llave, 2010, Silva et al., 2011). For instance, the *A. thaliana* and rice genomes each have four and eight DCLs, six and five RDRs, and ten and nineteen AGO proteins respectively, while *Populous* has five DCLs. *A. thaliana* possesses six silencing pathways (Brodersen and Voinnet, 2006, Margis et al., 2006). It has also been shown that in virus-infected *A. thaliana*, DCL4, DCL2 and DCL3 catalyze the formation of 21, 22 and 24 nt vsRNAs, respectively, to induce antiviral responses (Blevins et al., 2006, Ruiz-Ferrer and Voinnet, 2009). The DCL4 generated 21 nt and DCL2 generated 22 nt vsRNAs confer efficient antiviral defense in plants. Nevertheless, DCL4 and DCL2 exhibit functional redundancy or cooperative interaction since formation of the 22 nt vsRNAs mainly happens in the absence of DCL4 (Deleris et al., 2006, Donaire et al., 2008, Donaire et al., 2009, Ruiz-Ferrer and Voinnet, 2009). The 21 and 22 nt vsRNAs are the most predominant class amongst virus-infected host plants, with a few exceptions (Deleris et al., 2006, Ding, 2010, Garcia-Ruiz et al., 2010). Multiple AGO proteins are also involved in antiviral defense, including AGO2 and AGO5, which have been shown to bind cucumber mosaic virus (CMV)-derived small RNAs (Takeda et al., 2008). Similarly, RDR1, RDR2 and RDR6 have been implicated in vsRNA biogenesis and antiviral defense in plants (Ding, 2010, Ding and Voinnet, 2007, Donaire et al., 2008, Donaire et al., 2009, Garcia-Ruiz et al., 2010, Qi et al., 2009).

The biogenesis, composition, and abundance of vsRNAs have been characterized from several host–virus pathosystems (Donaire et al., 2008, Donaire et al., 2009, Lin et al., 2010, Miozzi et al., 2013, Naveed et al., 2014, Prabha et al., 2013, Silva et al., 2011, Visser et al., 2014, Xia et al., 2014). Small RNAs are known to play regulatory roles in defense responses against pathogens in plants (Ruiz-Ferrer and Voinnet, 2009, Seo et al., 2013). Different host plants have been shown to accumulate varying amounts of vsRNAs in response to infection by different viruses (Donaire et al., 2009, Lin et al., 2010). Through species diversity and epidemiology studies, CBSV and UCBSV have been shown to be closely related (Mbanzibwa et al., 2011, Mbanzibwa et al., 2009b). This has stimulated efforts to develop successful and durable CBSD resistance by simultaneous RNAi targeting of both viruses (Ogwok et al., 2012, Patil et al., 2011). However, no information is available concerning the biogenesis and composition of vsRNAs in CBSV- and UCBSV-infected cassava, nor their important, potential contribution to host plant resistance or susceptibility. The molecular mechanisms linking CBSV- and UCBSV-derived small RNAs to RNAi and CBSD symptom expression on different cassava genotypes is unclear. Alongside the premise that CBSV and UCBSV interact differently in cassava plants of the same genetic background (Ogwok et al., 2015), examining the CBSV- and UCBSV-cassava pathosystem offers a good platform to study molecular interactions between closely related RNA viruses in their natural host.

The present study aimed to characterize vsRNAs generated during separate CBSV and UCBSV infection in three cassava genotypes : NASE 3 (highly resistant to UCBSV but highly susceptible to CBSV), TME 204 (susceptible to both CBSV and UCBSV), and cultivar 60444 (highly susceptible to both CBSV and UCBSV). NASE 3 and TME 204 are farmer-preferred cassava varieties in Uganda (Taylor et al., 2012b), while 60444 is a model cassava genotype with well established tissue culture and transformation protocols, used to study gene expression including virus-derived transgenes tailored for resistance to CBSD viral pathogens (Bull et al., 2009, Vanderschuren et al., 2012, Yadav et al., 2011). The information generated is an important contribution for understanding the molecular mechanisms underlying the pathogenicity of CBSV and UCBSV and the response of some cassava genotypes to CBSD.

## 2 Materials and methods

### 2.1 Plant materials and virus inoculations

Micropropagated, tissue culture-derived cassava plants of NASE 3, TME 204 and 60444 (*N* = 20) were confirmed to be free of CBSD viral pathogens by reverse transcription polymerase chain reaction (RT-PCR) (Ogwok et al., 2012) and established in soilless compost as previously described (Taylor et al., 2012a). For each genotype, eight plants were bud-graft challenged at 10 weeks after planting (WAP) with CBSV Naliendele isolate (CBSV-[TZ:Nal3-1:07]) or UCBSV Kabanyoro isolate (UCBSV-[UG:T04-42:04]) as described by Wagaba et al. (2013). The grafted plants were assessed a week later for graft union formation and monitored visually thereafter for CBSD symptom development. CBSD symptom severity on grafted plants was scored using a scale of 1–5 as previously described (Yadav et al., 2011, Ogwok et al., 2012).

### 2.2 Sample collection, RNA extraction and detection of CBSV and UCBSV

Systemically infected leaves of cultivar 60444 and TME 204 showing clear CBSD symptoms, and stem sections of NASE 3 showing lesions typical of CBSD infection were collected six weeks after grafting (WAG). Similar tissues were sampled for use as controls from asymptomatic, non-inoculated plants of each genotype. Presence of CBSV and UCBSV was detected by RT-PCR and virus levels estimated by quantitative RT-PCR (qRT-PCR) as described previously (Ogwok et al., 2015). The plant materials were obtained from three independent biological replicates, wrapped with aluminum foil, immediately frozen in liquid nitrogen and stored at −80 °C. Total RNA was extracted from the resulting powder as previously described (Ogwok et al., 2012). The RNA was treated with DNase I (Ambion Inc., Austin, TX) according to manufacturer recommendations to eliminate genomic DNA contamination. RNA was quantified using a Nanodrop (Model 2000C, Thermo Scientific, Waltham, MA). Integrity of RNA with OD_260/280_ ratios from 1.8 to 2.1 was visually checked on 1.5% (w/v) agarose gel. Reverse transcription of RNA to cDNA was performed from 2 μg of total RNA using Superscript III™ First-Strand Synthesis System (Invitrogen, Carlsbad, CA) following manufacturer recommendations. An aliquot of the RNA was used to prepare small RNA libraries.

### 2.3 Small RNA library preparation

Small RNA libraries were prepared using the NEBNext^®^ Multiplex Small RNA Library Prep Set for Illumina^®^ (Sets 1 and 2) (New England Biolabs, Inc. Ipswich, MA) following manufacturer recommendations. Briefly, 3′ adapters were ligated to total RNA (6 μg) followed by hybridization of the reverse transcriptase primer to prevent adapter-dimer formation. Subsequently, 5′ adapters were ligated and the RNA subjected to reverse transcription and PCR amplification. The PCR products were purified using a QIAQuick PCR Purification Kit (Qiagen Inc., Valencia, CA) according to manufacturer recommendations and quality-checked by electrophoresis on a 6% polyacrylamide gel. Bands of ∼140 bp corresponding to the adapter-ligated small RNA fragments were isolated, crushed in elution buffer and re-precipitated using a mixture of Acrylamide, sodium acetate, and absolute ethanol. The small RNA libraries (cDNA pellet) were re-suspended in TE buffer (10 mM Tris–HCl containing 1 mM EDTA, pH 8) and re-checked for size and concentration by electrophoresis in 6% polyacrylamide gel.

### 2.4 Sequencing and bioinformatics analysis of small RNA sequences

The small RNA libraries were sent to the Genome Technology Access Center (GTAC), Washington University in St. Louis, Missouri, USA for further size, quality, and integrity checking by an Agilent 2100 Bioanalyzer (Agilent Technologies, Inc., Santa Clara, CA). Sequencing was performed by Illumina HiSeq 2,500 using 1 × 50 single-end read protocol. Raw sequence data received from GTAC was de-multiplexed by QIIME (Caporaso et al., 2010). Sequence reads with quality score below 19 were discarded. The bioinformatics tool Cutadapt (Martin, 2011) was used to remove adapter sequences. Small RNA sequences in the size range of 21–24 nt were selected for downstream analysis. The redundant as well as unique sRNA reads were mapped to the CBSV and UCBSV reference genome using Bowtie software (Langmead, 2010). Mapped reads data were converted to statistical data by BEDTools (Quinlan and Hall, 2010) and all outputs were graphically presented by Shell scripts provided by the Bioinformatics Core Facility at Donald Danforth Plant Science Center.

### 2.5 Quantification of DCL and AGO2 proteins by qRT-PCR

Quantitative RT-PCR (qRT-PCR) was performed to measure the expression levels of DCLs and AGO2 in CBSV- and UCBSV-infected cassava using cytochrome c oxidase (COX) mRNA as the reference gene for normalization as previously described (Ogwok et al., 2015). The primers used to amplify the DCLs and AGO2 are listed in Table 1. Briefly, cDNA was diluted ten-fold and subjected to qRT-PCR using BioRad CFX96Connect instrument (BioRad Laboratories Inc, Hercules, CA). Reaction mixtures contained 5 μl SsoFast Advanced SYBR Green I SuperMix, 1 μl of each primer (0.5 μM final concentration) and 3 μl of diluted cDNA template in a total reaction volume of 10 μl. The qRT-PCR thermal cycles used were as follows: initial denaturation step for 3 min at 95 °C, followed by 40 cycles of 10 s at 95 °C and 30 s at 58 °C. Data were collected at the end of each 58 °C cycle. Each qRT-PCR run included in each plate RNA that went through cDNA synthesis process with no reverse transcriptase enzyme added (NRT) and a no-template control (NTC) containing water instead of cDNA. The qRT-PCR was performed using three biological replicates for each sample and three technical replicates of each biological replicate. The mean quantification cycle (*C*_q_*)* value of each triplicate reaction was used for further calculations by the 2^−ΔΔCT^ method for relative normalized expression analysis using COX mRNA as reference gene, and CBSV or UCBSV positive samples of predetermined virus concentration as calibrator, respectively. The relative expression levels were obtained by comparing CBSV and UCBSV titers in virus-infected plants with uninfected plants.

## 3 Results

### 3.1 Response of cassava genotypes to infection by cassava brown streak viruses

The three cassava genotypes responded differently to graft-challenge with CBSV and UCBSV. All plants (*N* = 8) of genotypes TME 204 and 60444 grafted with CBSV-infected scions started showing typical CBSD symptoms two weeks after grafting, a week earlier than plants grafted with UCBSV-infected scions (Wagaba et al., 2013). NASE 3 plants grafted with UCBSV-infected buds obtained from either cultivar TME 204 or 60444 developed no symptoms, while plants of this cultivar grafted with CBSV-infected buds obtained from the same cultivars showed stem lesions around the graft union four WAG. Across all three genotypes, plants grafted with CBSV-infected buds showed more severe foliar CBSD symptoms compared to plants grafted with UCBSV-infected buds. CBSV-challenged plants of 60444, TME 204 and NASE 3 showed maximum mean severities of 4.3, 3.3 and 5.0, respectively, whereas UCBSV-challenged plants showed lower CBSD severities reaching maximum values of 3.0 and 2.0 for plants of 60444 and TME 204, respectively on a scale of 1–5 as previously described (Ogwok et al., 2012). As stated above, NASE 3 remained CBSD symptom-free after challenge with UCBSV (Table 2).

RT-PCR analysis revealed presence of CBSV in all CBSV-challenged plants showing CBSD symptoms across the three genotypes. qRT-PCR analysis showed that NASE 3 accumulated 2.7- and 1.4-fold higher CBSV RNA than 60444 and TME 204, respectively. Similarly, UCBSV was detected in all UCBSV-challenged plants of 60444 and TME 204, with 60444 found to have accumulated 1.4-fold higher UCBSV RNA than TME 204 (Supplementary Fig. S1). UCBSV was not detected by RT-PCR in plants of NASE 3 challenged with UCBSV. These results correlated with the visually assessed disease symptoms and indicated that CBSV was able to infect and accumulate viral RNA in all plants across genotypes, whereas UCBSV infected and accumulated viral RNA in TME 204 and 60444 but not in NASE 3 plants.

### 3.2 Deep sequencing of small RNAs in virus-challenged cassava genotypes

**Fig. 1:**
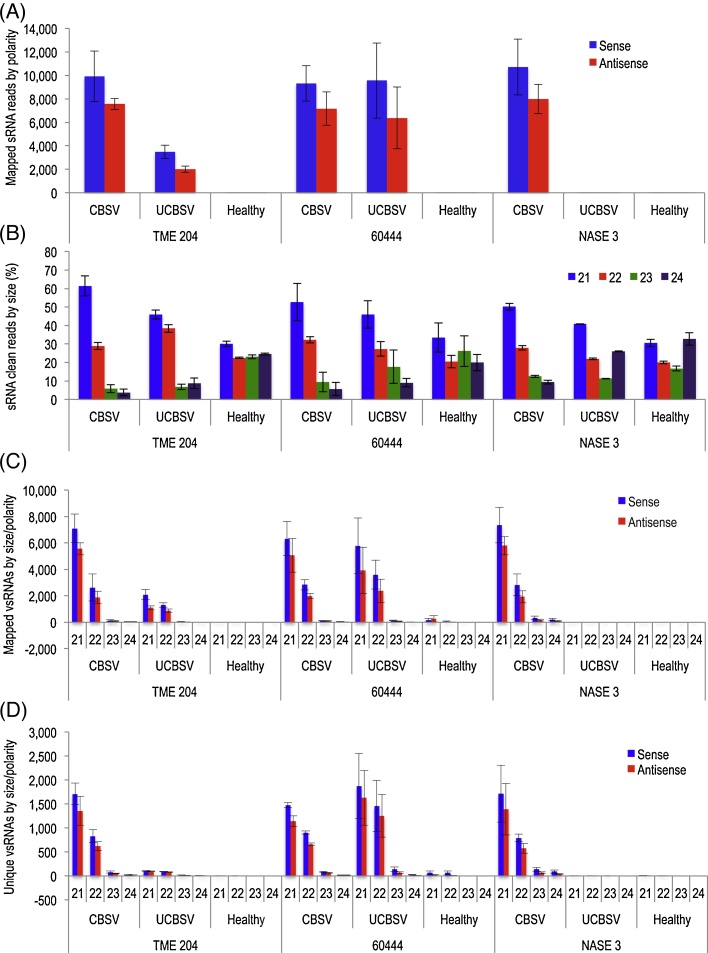
Comparison of CBSV- and UCBSV-derived small RNA populations recovered from infected and uninfected plants of TME 204, 60444 and NASE 3 (*n* = 3). The histograms represent (A) sRNA mapped to CBSV and UCBSV genomes in sense and antisense orientations, (B) Total sRNA sorted by percent nucleotide sequence lengths, (C) sRNA mapped to CBSV and UCBSV genomes, sorted by sequence lengths and orientations, and (D) unique (non-redundant) sRNA mapped to CBSV and UCBSV genomes, sorted by sequence lengths and orientations. Error bars indicate standard error of the mean (SEM).

Small RNA libraries for CBSV- and UCBSV-challenged and unchallenged cassava genotypes were prepared on Illumina platform. Sequence data showing total small RNAs obtained from leaves of uninfected and UCBSV- and CBSV-infected plants of TME 204, 60444 and NASE 3 are summarized in Table 2. The data presented is an average of three biological replicates per virus challenge for each genotype. The small RNA clean sequence reads (21 to 24 nucleotide adapter-trimmed sequence reads with Phred Quality score 20 and above) ranged from *c.* 10,000 to 1.3 million across libraries. The majority of small RNA reads were host derived. CBSV-derived small RNAs from cultivars TME 204, 60444 and NASE 3 challenged with CBSV accounted for up to 7%, 13% and 10%, respectively, of the total clean small RNA reads. Similarly, UCBSV-derived small RNAs in TME 204 and 60444 graft-inoculated with UCBSV accounted for 0.4% and 2.6%, respectively (Table 2). This contrasted with insignificant levels of UCBSV-derived small RNAs (0.002%) and UCBSV viral RNA accumulation obtained from UCBSV-challenged NASE 3 plants. The population of small RNAs in unchallenged control plants that mapped to viral genomes was minimal, accounting for 0.015%, 0.117% and 0.005% of total clean reads in TME 204, 60444 and NASE 3, respectively (Table 2; Fig. 1A).

Total small RNA reads identified from virus-infected plants were sorted according to their numbers and length. In all three cultivars, uninfected or infected with the virus, 21 nt total small RNAs were most abundant followed by 22 nt class. The 21 nt class was more abundant in virus-infected plants, representing 45–60% of small RNAs compared to 30–32% in uninfected plants (Fig. 1B). A similar trend was observed for 22 nt siRNAs. The proportion of 24 nt siRNAs was high in uninfected plants, accounting for 20–30% of siRNAs clean reads compared to <10% in virus-infected plants across genotypes. UCBSV-challenged and unchallenged plants of NASE 3 however accumulated similar levels (20–30%) of 24 nt siRNAs (Fig. 1B).

### 3.3 Characterization of CBSV- and UCBSV-derived small RNAs

To enable comparison of data across libraries, siRNA reads were normalized as reads per one hundred thousand of the total clean reads of corresponding samples. Sequences showing no mismatches were regarded as CBSV- or UCBSV-derived small RNAs, and each could be unambiguously assigned to one unique genome position. Small RNAs in the range of 21–24 nucleotides in size were included in this study. Analyses showed that the 21 nt size class was predominant (61–79%) followed by 22 nt size class (19–37%) for both viruses across the three cassava genotypes (Fig. 1C). Proportions of 23 and 24 nt classes were very low (1–2%; Fig. 1C). The vsRNAs of all size classes were mapped throughout the CBSV and UCBSV genomes in both sense and antisense orientations. A slight bias towards the sense polarity was observed, with 56–57% of the total CBSV-derived small RNAs and 60-63% of the total UCBSV-derived small RNAs of positive polarity. Similar results were obtained for unique vsRNAs (Fig. 1D), although the proportion of sense-unique UCBSV-derived small RNAs dropped to 53–54%.

**Fig. 2:**
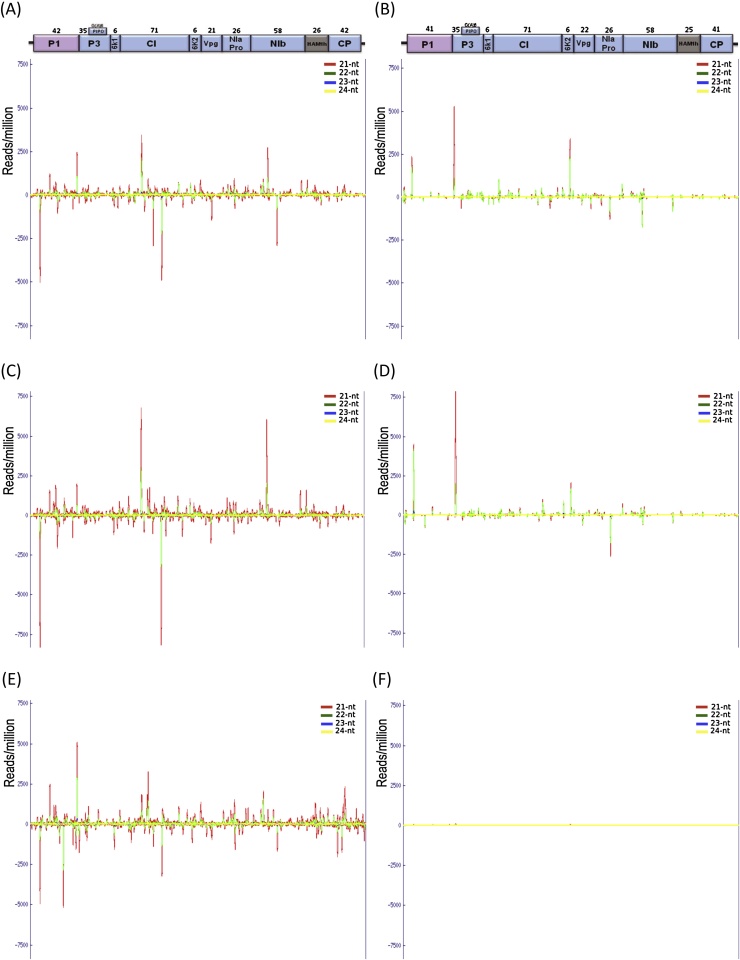
Genomic map of CBSV- and UCBSV-inoculated libraries. The graphs plot the number of 21–24 nucleotide virus-derived small RNAs at each position along the CBSV and UCBSV genomes recovered from (A) CBSV- and (B) UCBSV-inoculated TME 204; (C) CBSV- and (D) UCBSV-inoculated 60444; and (E) CBSV-inoculated NASE 3. Bars above and below the *x*-axis represent sense and antisense virus-derived small RNA reads, respectively.

**Fig. 3:**
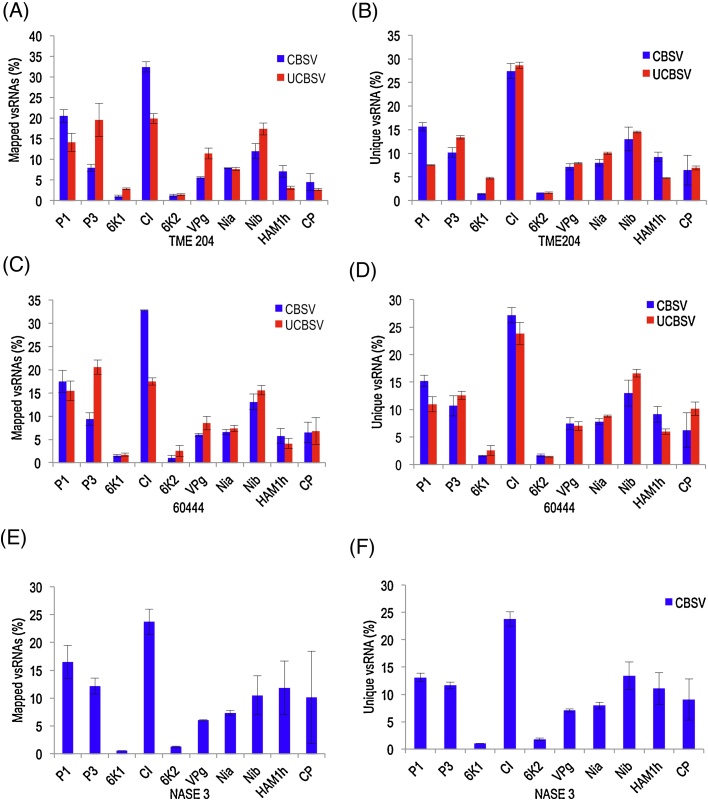
Total versus unique virus-derived small RNA population from individual genes of CBSV and UCBSV genomes in infected plants of TME 204, 60444 and NASE 3 (*n* = 3). The histograms represent (A) total mapped and (B) unique virus-derived small RNA in CBSV- and UCBSV-infected libraries of TME 204; (C) total mapped and (D) unique virus-derived small RNAs in CBSV and UCBSV-infected libraries of 60444; and (E) total mapped and (F) unique virus-derived small RNAs in CBSV-infected libraries of NASE 3. Error bars indicate standard error of the mean (SEM).

Different proportions of CBSV- and UCBSV-derived small RNAs were also reflected in line graphs obtained from normalized genome coverage data for each virus (Fig. 2A–F). The major classes of vsRNAs (21 and 22 nt sizes) were produced from all parts of the viral genomes across cassava genotypes. The distribution along the genomes were however non-homogenous. Some parts of the genomes were seen to be expressing more small RNAs than others. There was no correlation between percent of GC content (GC%) in different open reading frames (ORFs) of CBSV or UCBSV with vsRNAs. Examination of the percent vsRNAs for each genomic region showed that in CBSV-infected NASE 3 and CBSV- and UCBSV-infected 60444 and TME 204, the CI genomic region produced the highest population of vsRNAs, followed by genomic regions that encode NIb, P1, and P3 proteins, which produced moderate population of vsRNAs across viral genomes (Figs. 2 A–E; 3 A–F). The 6K1 and 6K2 genomic regions produced the lowest population of vsRNAs across viral genomes (Fig. 3A–F). Remarkably, profiles of CBSV- and UCBSV-derived sRNAs were different in the same cassava genotype, but similar for each virus across genotypes, except for UCBSV-challenged NASE 3 (Fig. 2F).

**Fig. 4:**
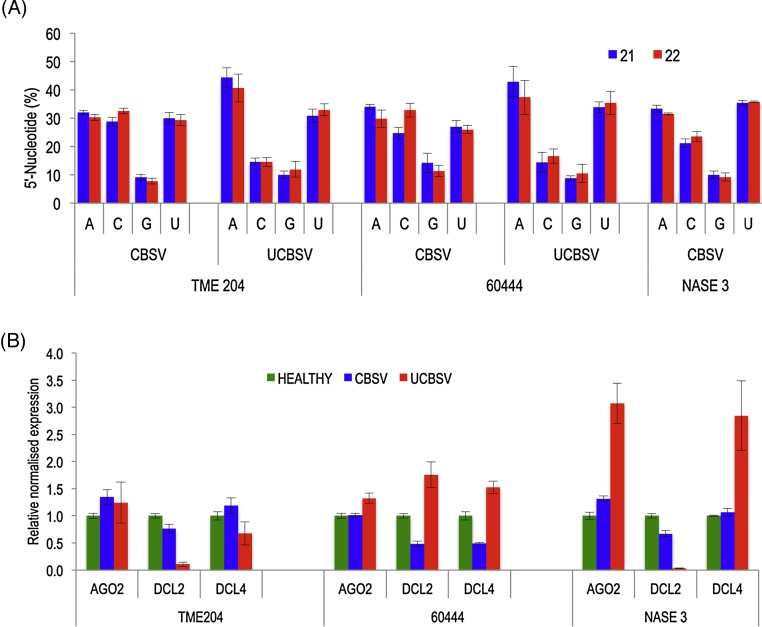
Relative frequency of first 5′-end nucleotide and relative expression levels of Dicer and Argonaute proteins mRNAs. (A) The histograms represent relative abundance of the four distinct nucleotides at the 5′-end of 21 and 22 nucleotide sized virus-derived small RNAs of CBSV- and UCBSV-infected libraries of TME 204, 60444 and NASE 3 (*n* = 3). (B) Real-time PCR analysis of the expression levels of cassava homologues of AGO2, DCL2 and DCL4 in CBSV- and UCBSV-infected and uninfected plants of TME 204, 60444 and NASE 3 (*n* = 3). Error bars indicate standard error of the mean (SEM).

In *A. thaliana*, sorting of small RNAs into Argonaute complexes is directed by the first 5′ nucleotide (Mi et al., 2008). To predict selective interaction of vsRNAs with specific AGOs, the relative abundance of total vsRNAs was determined based on the first 5′-terminal nucleotides. Small RNAs with G as the first nucleotide at 5′ end were the least abundant (8–11%) for both viruses across all three genotypes (Fig. 4A). In UCBSV-infected TME 204 and 60444, the majority of UCBSV-derived small RNAs had A (40–45%) followed by U (30-34%) as the most abundant first nucleotide at 5′ end. In CBSV-challenged TME 204, 60444 and NASE 3, CBSV-derived small RNAs starting with A (32–34%), U (30–35%) and C (20–30%) at 5′ end were all in similar proportions.

### 3.4 Distribution and frequency of vsRNAs along CBSV and UCBSV genomes

Small RNA mapping analysis identified four major hotspots rich in 21 and 22 nt vsRNAs in each of the CBSV and UCBSV genomes (Fig. 2A–E). In the CBSV genome, the first hotspot was found within the cylindrical inclusion protein (CI) region from 2216 to 4105 nt position that correlated with 32–33% of all the CBSV-derived small RNAs produced from this region in TME 204 and 60444 (Fig. 3A & C). In NASE 3, only about 24% of CBSV-derived small RNAs were produced from this region (Fig. 3E). A hotspot was also found in the same region (from 2209 to 4092 nt) in the UCBSV genome, and was responsible for 17-20% of UCBSV-derived small RNAs (Fig. 3A and C). A second hotspot was located in the large nuclear inclusion protein (NIb) region from 5522 to 7026 nt in the CBSV, and 5506 to 7011 nt in the UCBSV genome. These hotspots produced 11-13% CBSV-derived small RNAs and 16–19% UCBSV-derived small RNAs in the CBSV and UCBSV genomes, respectively (Fig. 3A, C and E). The third and fourth hotspots were found located in the P1 and P3 genomic regions. The P1 region in the CBSV genome (104–1177 nt) yielded 16–20% CBSV-derived small RNAs, whereas the P3 region (1178–2059 nt) produced 8–12% CBSV-derived small RNAs. In the UCBSV genome, the P1 region (85–1170 nt) produced 14–18% of UCBSV-derived small RNAs whereas the P3 region (1171–2052 nt) produced 19–20% of UCBSV-derived small RNAs (Fig. 3A and C). Similar trends were obtained for unique vsRNAs across genotypes (Fig. 3B,D,F).

### 3.5 Expression of DCL and AGO2 proteins in CBSV- and UCBSV-infected cassava

Dicer-like proteins play a major role in the production of small RNAs. To understand the effect of CBSV and UCBSV infection on RNA silencing pathways, qRT-PCR analysis was performed to determine transcript levels of cassava homologues of DCL (*Manihot esculenta* DCL) proteins and their correlation to the abundance of 21 and 22 nt vsRNAs in CBSV- and UCBSV-infected plants. Differing patterns of expression for these genes were observed across the three cassava genotypes when they were infected with the two viral pathogens. In CBSV-infected TME 204, there was down-regulation of *MeDCL2* mRNA and 1.2-fold up-regulation of *MeDCL4* mRNA compared to the uninfected control. In UCBSV-infected TME 204, both *MeDCL2* and *MeDCL4* transcript levels were downregulated. In CBSV-infected 60444, both *MeDCL2* and *MeDCL4* transcript levels were downregulated, whereas in UCBSV-infected 60444 plants both *MeDCL2* and *MeDCL4* mRNA levels were upregulated (Table 2, Fig. 4B). In NASE 3, *MeDCL2* mRNA level was downregulated in both CBSV- and UCBSV-challenged plants compared to unchallenged controls. In contrast, *MeDCL4* mRNA was upregulated in both CBSV- and UCBSV-challenged plants compared to unchallenged controls (Table 2; Fig. 4B).

Multiple AGO-bound small RNAs guide effector complexes to the target viral mRNAs in a sequence-specific manner leading to either translational repression or mRNA cleavage (Ding, 2010, Ding and Voinnet, 2007, Llave, 2010). The vsRNAs, dictated by their first 5′-end nucleotides, are preferentially sorted and loaded into multiple AGO complexes (Mi et al., 2008, Takeda et al., 2008). Specifically, AGO1 has been shown to have preference for U, AGO2 and AGO4 have preference for A or U, while AGO5 prefers C at the first 5′-end of the siRNA (Donaire et al., 2009, Mi et al., 2008, Qi et al., 2009, Takeda et al., 2008). Since in this study a major proportion of vsRNAs have A or U at the first 5′ end, accumulation of cassava homologue of AGO2 (*MeAGO2*) mRNAs was determined by qRT-PCR. The results showed that *MeAGO2* mRNA was upregulated in all three UCBSV-infected cassava genotypes and CBSV-infected TME 204 and NASE 3 plants. However, MeAGO2 expression remained unchanged in CBSV-infected 60444 (Table 2; Fig. 4B).

## 4 Discussion

To decipher the molecular mechanism and RNAi components involved in CBSV and UCBSV infection, three cassava genotypes TME 204 (CBSD susceptible), 60444 (CBSD susceptible) and NASE 3 (CBSD tolerant) (Abaca et al., 2013) were challenged with CBSV and UCBSV, and the population and characteristics of sRNAs studied by next generation sequencing. Sequence analysis showed that populations of pathogen-derived siRNA varied across genotypes. Maximum populations of small RNAs that mapped to the virus genome were found in CBSV-infected plants of 60444 (12.7%) followed by NASE (9.9%) and TME 204 (7.3%), respectively. In UCBSV-infected plants, maximum populations of UCBSV-derived sRNAs were found in 60444 (2.6%) whereas 0.4% of UCBSV-derived sRNAs accumulated in TME 204. In NASE 3, UCBSV-derived sRNAs were insignificant (0.002).

Quantitative RT-PCR analysis also showed that NASE 3 accumulated 2.7- and 1.4-fold higher CBSV RNA than CBSV-infected plants of 60444 and TME 204, respectively (Fig. S1). Similarly, UCBSV-infected 60444 accumulated 1.4-fold more UCBSV RNA than UCBSV-infected TME 204 (Fig. S1). Comparable qRT-PCR data was obtained in a previous study using field and glasshouse samples from the same cassava genotypes (Ogwok et al., 2015). Data presented here for the lack of visual symptom development and non-detectable presence of the virus confirms that observation that UCBSV does not infect NASE 3 plants (Table 2). The variation in cultivar responses could be due to their ability to differentially recognize CBSV and UCBSV and accordingly trigger antiviral defense mechanisms, or it could be due to inherent differences in the infection cycle of the two viruses including viral replication and accumulation of vsRNAs (Naveed et al., 2014). Hitherto, there was a positive correlation between virus titer and the levels of vsRNAs across genotypes. Susceptible cassava genotypes (TME 204, 60444 and NASE 3 for CBSV, and TME 204 and 60444 for UCBSV) accumulated high virus titer and high levels of vsRNAs. We suggest that the high accumulation of vsRNAs observed in susceptible genotypes may be due to failure to target virus genome resulting in continued virus replication and symptom persistence. In contrast, NASE 3, resistant to UCBSV, accumulated negligible UCBSV mRNA and insignificant levels of UCBSV-derived sRNAs. This may be due to efficient PTGS of viral mRNA, leading to a depletion of vsRNA populations.

CBSV or UCBSV infection was shown here to alter host small RNA profiles across the three cassava genotypes studied. The levels of 23 and 24 nt size classes were high in uninfected libraries but were less abundant in virus-infected libraries across all genotypes. The 21 and 22 nt siRNAs were abundant in uninfected plants but were even higher in virus-infected plants across genotypes. Similar studies in uninfected *A. thaliana* showed that the 24 nt siRNAs were most abundant (35%), followed by 21 nt siRNAs (28%), but upon infection with cabbage leaf curl virus (CaLCuV), the host siRNA profile was altered such that the 21 nt class became more abundant (32%) followed by the 24 nt class (28%) (Aregger et al., 2012). Also, in sugarcane mosaic virus (SCMV)-infected maize, there was increased level of 21 and 22 nt size classes, whereas that of 24 nt size class decreased (Xia et al., 2014). Contrastingly, cauliflower mosaic virus (CaMV)-infected *A. thaliana* resulted in overaccumulation of the 24 nt siRNAs (Blevins et al., 2011).

Reports in *A. thaliana* have shown that DCL4, DCL2 and DCL3 generate the 21, 22, and 24 nt vsRNAs (Blevins et al., 2006, Donaire et al., 2008, Ruiz-Ferrer and Voinnet, 2009). In addition, studies in *A. thaliana* infected with turnip mosaic virus (TuMV), crucifer-infecting strain of tobacco mosaic virus (TMV-Cg), cucumber mosaic virus (CMV), and tobacco rattle virus (TRV) (Donaire et al., 2008, Donaire et al., 2009, Lin et al., 2010, Qi et al., 2009) and *Nicotiana benthamiana* infected with potato virus x (PVX),bamboo mosaic virus (BMV), and pepper mild mottle virus (PMMoV) (Donaire et al., 2009, Lin et al., 2010); potato infected with three different strains of potato virus y (PVY-O, PVY-N, and PVY-NTN) (Naveed et al., 2014); grapevines infected with grapevine fleck virus (GFkV) and grapevine rupestris stem-pitting associated virus (GRSPaV) (Pantaleo et al., 2010); cotton infected with cotton leaf roll dwarf virus (CLRDV) (Silva et al., 2011); and maize infected with SCMV (Xia et al., 2014), among others, revealed that the majority of vsRNAs in infected plants belonged to the 21 and 22 nt size classes, which are associated with activated PTGS (Blevins et al., 2006, Deleris et al., 2006, Donaire et al., 2008). Similarly, the 21 and 22 nt CBSV- and UCBSV-derived sRNAs were shown here to be the most predominant, which suggests activity of homologues of DCL4 and DCL2 in response to virus infection in cassava (Fig. 4B). The low abundance of the 23 and 24 nt vsRNAs in both CBSV- and UCBSV-infected plants across cassava genotypes suggests marginal activity of DCL3 in cassava against these viruses (Qi et al., 2009).

DCL2 and DCL4 proteins are key components of RNAi pathways and both are required for optimal resistance against viruses. Despite differences in the silencing activity of their small RNA products, DCL2 and DCL4 mostly act redundantly yet hierarchically when present simultaneously (Parent et al., 2015). Quantitative PCR analysis of DCL2 and DCL4 expression in this study showed that both proteins have different expression patterns in response to CBSV and UCBSV infection. Particularly, in NASE 3 challenged with either CBSV or UCBSV and TME 204 challenged with CBSV, DCL4 was up-regulated whereas DCL2 was down-regulated. A similar observation was reported in CLRDV-infected cotton (Silva et al., 2011). Presumably an up-regulation of DCL4 is sufficient to suppress UCBSV infection in NASE 3 plants. A detailed study of both DCL2 and DCL4 in a background of two different host genotypes would be of great interest.

Processing of dsRNAs by Dicer proteins would ideally yield an equal amount of sense and antisense vsRNAs, the life span of which depends on selective incorporation into specific AGO proteins (Qi et al., 2009). Analysis of the CBSV- and UCBSV-derived small RNA polarity showed a bias towards the sense polarity compared to antisense polarity irrespective of size class across libraries, and was consistent with previous findings in other virus-infected plants (Donaire et al., 2008, Donaire et al., 2009, Naveed et al., 2014, Qi et al., 2009). Strand biases are usually attributed to preferential processing of highly structured single-stranded genomic viral RNAs by Dicer proteins (Ding and Voinnet, 2007, Donaire et al., 2009, Silva et al., 2011), and different viruses have been shown to produce, in the same host plant, virus-derived small RNAs with different ratios of sense to antisense polarity (Donaire et al., 2009, Pantaleo et al., 2010). However, a correlation between vsRNA hotspots and structured regions of genomic viral RNAs remains unclear (Donaire et al., 2009).

The preferential use of vsRNAs with A or U residues as compared to C and G residues as the first 5′-end nucleotide has been reported in *A. thaliana* infected with TuMV and PVX, *Cucumis melo* infected with WMV (Donaire et al., 2009), and in potato plants infected with three strains of PVY (Naveed et al., 2014). In contrast, a few cases of preferential use of C as the first 5′-terminal nucleotide has been reported in grapevines infected with GFkV and GRSPaV (Pantaleo et al., 2010), and tomato plants infected with tomato yellow leaf curl Sardinia virus (TYLCSV) (Miozzi et al., 2013). However, most previous studies reported a tendency to avoid vsRNAs with G residues at the first 5′-end (Donaire et al., 2009, Mi et al., 2008, Pantaleo et al., 2010), probably due to absence of AGO proteins with known preference for G residues at the first 5′-end (Mi et al., 2008). Our results indicate that A and U were the most abundant nucleotides at the first 5′-end (∼80%), while G was the least abundant at the first 5′-end (Fig. 4A). In addition, there was up-regulation of AGO2 mRNA in all three UCBSV-infected cassava genotypes and CBSV-infected TME 204 and NASE 3 (Fig. S1). Similar studies in turnip crinkle virus (TCV)- and CMV-infected AGO2 mutant *A. thaliana* compared to wild type plants revealed the mutants were highly susceptible to TCV and CMV infection (Harvey et al., 2011). Besides, the induction of susceptibility involved activity of viral silencing suppressor proteins (Endres et al., 2010, Lewsey et al., 2010). Our results signify increased activity of AGO2 homologue in CBSV- and UCBSV-infected cassava. However, abundance of A, U and C at the first 5′ terminus in similar proportions in CBSV-infected plants imply the involvement of multiple AGOs in sorting out the vsRNAs.

Host RDRs use viral ssRNA to synthesize dsRNAs, which serve as substrates for DCL-dependent formation of secondary vsRNAs to maintain systemic silencing throughout the plant. In addition, DCL4 and RDR1 were reported as major contributors to the abundant pool of 21 nt TuMV-derived siRNAs in *A. thaliana* (Garcia-Ruiz et al., 2010). However, the response of RDRs in CBSV- and UCBSV-infected cassava was not assessed in this study. Since ipomoviruses and potyviruses (TuMV) share many characteristics, such a study is worth considering.

Furthermore, viral suppressor proteins have been shown to employ a multitude of mechanisms (Csorba et al., 2015, Ding and Voinnet, 2007). For example, the P0 protein of TCV has been shown to target AGO1, leading to its degradation though it does not interfere with siRNA-RISC assembly. Similarly, potyviral HC-Pro and ipomoviral P1b protein of cucumber vein yellowing virus (CVYV) have been shown to suppress plant silencing machinery through siRNA sequestration thereby interfering with viral RNA degradation (Valli et al., 2007). In addition, although the ipomoviruses sweet potato mild mottle virus (SPMMV) and tomato mild mottle virus (TomMMV) both encode HC-Pro, which is related to potyviral HC-Pro, the HC-Pro of SPMMV lacks RNA-silencing suppressor activity. Instead, the P1 protein acts as the RNA-silencing suppressor by binding with AGO1 thereby interrupting RISC assembly (Dombrovsky et al., 2014). The function of the CBSV or UCBSV P1 protein, a putative RNA silencing suppressor (Mbanzibwa et al., 2009a) remains a subject for further study. This could provide insights into the observed disparity between CBSV and UCBSV pathogenicity.

To our knowledge, this study provides the first high-resolution genome map of vsRNAs for an ipomovirus in the family *Potyviridae*. The use of deep sequencing in this study has provided an insight into the molecular interaction between CBSD causing viruses and cassava. The populations of vsRNAs were abundant, diverse and revealed widespread targeting of viral genomes by machinery of the gene silencing pathway. The overall composition of sRNAs in virus-infected cassava unveiled the action of different Dicer proteins in different cassava genotypes. The findings also provided an insight into the differential susceptibility of host plants to the same virus, which is reflected in the severity of symptoms they induce. Finally the results indicate that CBSV and UCBSV interact differently in the same host genetic background.

## Conflict of interest

The authors have no conflict of interest to declare.

## Figures and Tables

**Table 1:** Primer sequences and amplicon sizes of cassava homologues of Argonaute 2 (AGO2), and Dicer-like proteins 2 and 4 (DCL2 and DCL4).

Gene	Primer code	Primer sequence	Amplicon size (bp)	Efficiency (%)	*R*^2^	Transcript ID
AGO2	AGO2-F1	GGCAATCTCCAGCTTCAGCA	78	104.5	0.993	cassava4.1_000920 m
AGO2-R1	TCCAATGAAGCAGCCGATGA
DCL2	DCL2-F3	ATGCACACTGACCTCGTC	110	99.1	0.986	cassava4.1_000931m
DCL2-R3	GTCATCACAAGCACCTCA
DCL4	DCL4-F3	TGCTACTAAAGTGGGTGAAGAAG	109	106.8	0.977	cassava4.1_001038m
DCL4-R3	CGCACGTCCTCTAGATGGTATG

**Table 2:** Summary of small RNA reads from two cassava brown streak viruses in three different cassava genotypes^a^ and effect of the viruses on AGO2, DCL2 and DCL4 expression.

Genotype	Virus species	Mean CBSD severity 6 WAG (1–5)^b^	Total raw reads	Total clean reads	Small RNAs mapped to virus genome	Percent vsRNAs with respect to total clean reads	Effect of virus on AGO2, DCL2 and DCL4 expression		
AGO2	DCL2	DCL4
TME204	CBSV	3.3	3,185,175	100,750	17,470	7.340	up	down	up
UCBSV	2.0	3,994,736	1,282,370	5,488	0.428	up	down	down
Healthy	1.0	2,740,771	86,524	13	0.015	na	na	na
60444	CBSV	4.3	3,198,984	130,400	16,496	12.650	na	down	down
UCBSV	3.0	4,020,006	612,987	15,939	2.600	up	up	up
Healthy	1.0	3,347,340	10,238	12	0.117	na	na	na
NASE3	CBSV	5.0	3,019,728	188,617	18,705	9.917	up	down	up
UCBSV	1.0	2,511,404	410,641	10	0.002	up	down	up
Healthy	1.0	2,154,804	213,163	10	0.005	na	na	na

a Data presented are an average of three biological replicates per genotype.

b CBSD symptoms on grafted plants were scored as previously described (Ogwok et al., 2012). na = not applicable.
