# Supplementary figures and images for: Comparative analysis of virus-derived small RNAs within cassava (Manihot esculenta Crantz) infected with cassava brown streak viruses
Source: Virus Res. 2016 Apr 2;215:1–11. doi: 10.1016/j.virusres.2016.01.015 (PMC4796025; doi:10.1016/j.virusres.2016.01.015)

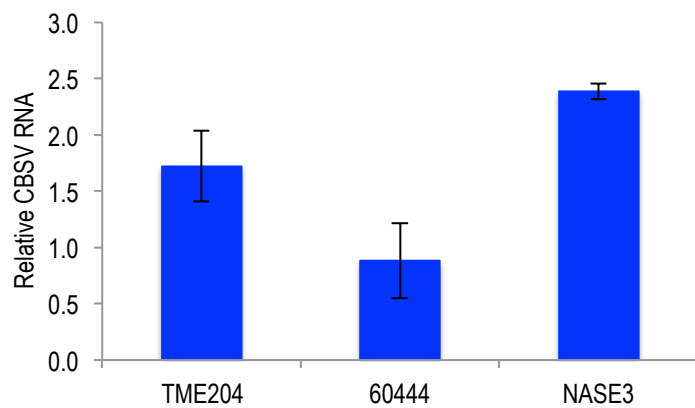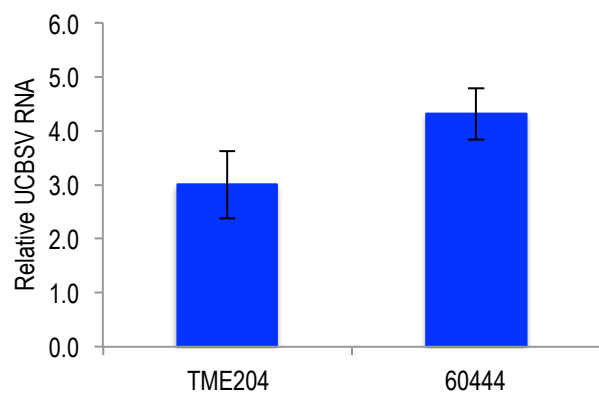

Supplement: Supplementary file 1 [file mmc1.pdf]
